# Practical Medicine

**Published:** 1878-07

**Authors:** 


### Practical Medicine.

Contagiousness of Phthisis. — Tappenier ("*Lo Sperimentale*,") Jan., 1878.

All physicians have observed cases of phthisis rapidly developed in individuals who had for a long time attended on patients in this disease, even when such attendants had not presented any predisposition, either individual or hereditary. Dr Tappenier believes that the explanation of the fact is to be found in the inhalation of the expectorated matter, scattered in the air by the coughing of patients. In order to test this opinion, he made experiments, by intimately mixing a certain quantity of the sputa in a little water; he pulverized this emulsion by an appropriate process, and subjected some animals to the inhalation of the substance during one or two hours every day. These experiments were made in the Anatomico-Pathological Institute of Prof. Buhl of Monaco. Dogs were selected, as animals presenting the least predisposition to contraction of the disease. Three perfectly sound dogs were put into the pen of the institute; the pen is situate near a window, and is closed in all parts, excepting above, where it receives the external air through a door which is furnished with a fastening. Some sputa was obtained from a patient in phthisis, a spoonful of which was mixed in a quantity of water sufficient to make of it a liquid similar to almond milk, and every day pulverization of this was made in the pen during an hour, or an hour and a half. At the same time. for the purpose of studying absorption, by the digestive system, of the tuberculous matter, two of the dogs were made to swallow a certain quantity of it, from the same patient.

The whole five dogs had apparently a good appetite, and presented neither cough nor diarrhœa, they ate freely, and were cheerful and nimble, without any symptoms of illness, unless a slight wasting and arrest of development. At first view, therefore, the experiments gave a negative result. But the day preceding the first autopsy, a little finely powdered carmine was mixed with the tuberculous liquid, in order to discover how far it had penetrated into the respiratory passages. Two of the dogs subjected to inhalation, and the two which had swallowed the tuberculous mixture, were killed six weeks after the commencement of the experiment. The autopsy of the fifth had been made at the end of three weeks.

The results of the autopsies were surprising. The five dogs presented a general miliary tuberculosis of both lungs, of the liver, the kidneys, and (at least in the two that had swallowed the tuberculous matter) of the digestive apparatus. The numerous stains of carmine which were seen on the pulmonary surface, showed that the inhaled liquid had penetrated into the pulmonary cells. The microscopic examination made by Prof. Buhl, established in the clearest manner the reality of the lesions.

It has, therefore, been established experimentally that in the dog, a general miliary tuberculosis can be induced from the inhalation, or the ingestion of the matter expectorated by a phthisical patient. The possibility of contagion of phthisis through the natural channels, may therefore be concluded.

The hygienic and clinical consequences of the experiment are of high importance. And first of all it is to be noted that those dogs continued in apparent sound health, despite the existence of general miliary tuberculosis. It is, therefore, possible that in man a miliary tuberculosis may rest latent during a certain time, and may not become a real and declared phthisis, before the development of foci of inflammation. But that which is of chief importance is the possibility of transmission of tuberculosis from man to man.

In ordinary conditions, that is to say, in fresh and frequently renewed air, the matters expectorated, and suspended in the air, may not become sufficiently concentrated to have the power of inducing tuberculous infection. But when a certain number of phthisical patients reside together, and through fear of cold or of draughts, the place is but little, or not at all ventilated, may we not fear that the expectorated matter will accumulate sufficiently to become dangerous to healthy persons living in the same quarters? Ought we not, therefore, in this regard, to take precautions, sometimes neglected, particularly in the wards of hospitals? Is it not, perhaps, prudent to recommend to consumptives never to swallow the matter brought up from cavities, which may have a deleterious influence on the digestive canal? Finally, may not these experiments, in some degree, explain the transmission of phthisis from husband to wife, or *vice versa*, and, consequently the advisability of avoiding conjugal intercourse?

The facts stated by Dr. Tappenier are of great interest, and may explain many points of the important question of the contagion of phthisis.

THE PREVENTION OF PUERPERAL FEVER. (*Berliner Klin. Wochenschrift*, 1878.)

It appears that the idea of "listering" in obstetrics (the Germans have coined the verb "listern" to express the use of Professor Lister's antiseptic method, just as from Galvani's name we have coined the verb "galvanize") was first started by Bischoff, of Basle, in 1870 (*Correspondenzblatt für Schweizer-Aertze*, 1875, Nos. 22, 23). His plan consisted in giving a bath as soon as the first pains of labor were observed, washing out the vagina with a 2 per cent. solution of carbolic acid every two hours, and anointing the fingers of the medical attendant with 10 per cent. carbolic oil at each examination, the hands being previously disinfected by washing them with 3 per cent. aqueous carbolic acid. In case the hand had to be passed into the uterus, or if the fœtus was dead and decomposed, the uterus was washed out with a 2 to 3 per cent. solution of carbolic acid; and in every case frequent injections of the latter were made into the vagina and uterus for thirteen days after the birth of the child. Immediately after the labor, any wound was touched with a 10 per cent. carbolic solution, no ligature, if such were necessary, being applied until this had been done. Lastly, a pad of wadding, soaked in carbolic oil (one to ten), was placed in the entrance of the vagina, and constantly renewed. Under this system the number of cases in which morbid symptoms were present, consisting in a febrile temperature of more than two days' duration, and reaching 38.5°

Cent. (101.3° Fahr.) at least on one day, tenderness of the abdomen on pressure, fetid discharge, etc., was, in 1870, 14 per cent.; 1871, 22.3 per cent.; 1872, 24.5 per cent.; 1873, 16.8 per cent.; 1874, 10.7 per cent.; 1875, 8.9 per cent.; or taking the average of the whole, 16.2 per cent. for the six years.

In 1875, H. Fehling published (*Archiv für Gynakologie*, Band xiii., s. 298) the results of experiments made for about a year in Professor Credé's clinic at Leipsic, and which consisted in applying a mixture of salicylic acid and starch (one to five) to any wounds of the external genitals and in syringing the vagina four to eight times daily, in case of fever and fetid discharge, with solutions of salicylic acid ($\frac{1}{6}$ to $\frac{1}{10}$ per cent.) The effect was excellent, but the use of the carbolic spray during labor, which was also tried for some time, was given up in consequence of the post-partum hemorrhages which it appeared to induce.

In 1877, Adrian Schücking (*Berliner Klin. Wochenschrift*, No. 26) suggested that the vagina should be washed out at the end of the labor with a 5 per cent. carbolic solution, and that immediately afterwards the uterus should be continuously irrigated by means of the apparatus of which we gave a brief description in our former article on this subject. This method was carried out in eight cases, in five of which the patients had had severe labors, and all recovered satisfactorily, no temperature being recorded over 38.4° Cent. In the other three, the injection was not begun until after the commencement of febrile symptoms, but an immediate and decided defervescence was the result. Professor Zweifel's objection to Schücking's conclusion, that in the five former cases the fortunate termination was directly due to the treatment, is, first, that the number of Schücking's cases is too small; and secondly, that equally good results are possible without any antiseptic treatment. With this objection most persons will, we think, be inclined to agree.

Professor Zweifel's own method, to which we shall devote the remainder of this article, is founded partly on the use of antiseptic measures, properly speaking, and partly on the adoption of the most scrupulous cleanliness in connection with the surroundings of the puerperal woman. In the first place, all vaginal examinations *during pregnancy* are, in his clinic, made only after careful washing of the hands and smearing with carbolic oil, the vagina being further washed out afterwards in some cases with 5 per cent. carbolic solution. The reason for these precautions is the possibility of infectious matter being introduced into the vagina previous to labor, of its lying there and being sucked up into the uterus after the expulsion of the fœtus. "This," says Professor Zweifel, "is a possibility which no one will deny."

The rooms and beds destined for the use of the lying-in women are carefully disinfected by burning sulphur in them in fireproof vessels, allowing about four grammes of sulphur to each cubic metre of space. The bedclothes are spread out so as to expose as large a surface as possible to the fumes, which after a few hours are allowed to escape by opening the windows.

After each labor in which the hand has been introduced into the uterus, or where air has gained entrance to it, or gaseous decomposition occurred in it, the uterus is washed out with several litres of fresh water.

Since almost all the cases of puerperal fever are found to be complicated either with ruptured perineum, small rents in the vagina and vulva, or with the introduction of air into the uterus during some operation, the greatest care is bestowed on all external wounds, to which Fehling's mixture of salicylic acid and starch is applied with the best results. Careful examination of the external genitals day by day, and the use of the thermometer, are also rigorously attended to. It should be added that, at Erlangen, the Obstetric Clinic has a separate pavilion to itself, which was built in 1874. The number of births from April, 1876, to October, 1877, during which period the above method has been carried out "with pedantic strictness," has been 184, with a *single* death—that of a woman with cancer, on whom the Cæsarean operation was performed. In 143 cases the lying-in period was completely normal—that is to say, the temperature never exceeded 38° Cent., or, at any rate, was never above 38.4° on more than one day. Out of the remaining forty-one, thirteen never had any morbid symptom except a rise of temperature on one or two days to 38° to 39° Cent., or on several days to 38° to 38.5°; twenty-eight had the symptoms of puerperal fever in a greater or less degree, but of these only twelve had protracted fever, inflammatory exudation, and showed clear signs of puerperal infection, and in only five cases was life ever in any apparent danger. It was further noticed that the cases which did badly were not evenly distributed through the whole period of observation, but were limited to the months of December, 1876, and January, 1877, and of September and October, 1877, in the form of small epidemics. On the whole, Professor Zweifel considers that his results are by no means inferior to those of Bischoff, and that they do not point to any necessity for introducing a more complicated antiseptic system into his practice. Moreover, Spiegelberg at Breslau, has carried out a system closely resembling Zweifel's since 1874, with the splendid result of only *five* deaths in *nine hundred* labors.*

With such evidence before us it seems to be our bounden duty to urge on the medical profession in this country to habitually adopt the measures by which alone, as far as present knowledge goes, puerperal infection can be prevented—namely, scrupulous cleanliness and the use of antiseptic lotions, etc., for disinfecting the examining hand and the genital organs. Even the busiest practitioner can manage to invariably examine with carbolic oil instead of ordinary oil or grease, and in the most out-of-the-way places vinegar or brandy, as Professor Zweifel says, are sure to be found as substitutes for carbolic or salicylic acid.

We are not sure that in private practice the need of these precautions is not as great as in the hospital ward; for the risk of picking up infection somewhere, and conveying it to the lying-in room, is naturally very great when the same man is seeing on the same day medical, surgical, and obstetric cases. He may go straight from a scarlet fever case to a woman in labor; and a most melancholy instance occurs to us in which a very valuable life was probably sacrificed in this way not so very long ago. The old discussions about puerperal fever, which we find reproduced even now in text-books on midwifery, are out of date in the light of our modern knowledge. We *know*, for example, that the woman who gets fever, peritonitis, and vomiting just

* For further information on this subject see also the *Zeitschrift f. Gerburtshülfe und Gynækologie*, ii., 1, containing papers by Schülien, Richter, and Langenbuch.

after her confinement, has been infected with poison *from without*—whether bacterial or otherwise makes not the slightest difference; we *know*, too, how to prevent the entrance of this poison into the woman's system, though we may be very helpless when it has once entered it. Knowing all this, and knowing, too, the high mortality from puerperal fever, and that probably more than a thousand women die of it in England every year, is it not our plain and simple duty to try and carry out, at any rate, the major operations of midwifery in future, with the same attention to antiseptic precautions as Mr. Spencer Wells observes in performing ovariotomy?

Œdema of the Lungs.—In a lengthy article in Virchow's Archiv (Bd 72, Heft. iii.), Dr. W. H. Welch, of New York, gives the results of his experiments conducted in Cohnheim's laboratory, on the causes of œdema of the lungs. Leaving aside the collateral and inflammatory forms, which he refers to inflammatory alteration of the vessel, he made a special study of congestive œdema. In the first place, attempts were made to cause pulmonary congestion (and œdema) by obstructing the aorta and its branches. In the rabbit œdema could be obtained only by ligating all vessels carrying blood from the left ventricle, except one—the carotid or subclavian artery. In the dog, it was found absolutely necessary to close the aorta and all its branches.

Lichtheim had previously found—and this was confirmed by Welch—that the tension in the pulmonary arteries is remarkably independent of the blood-pressure in the systemic circulation, a ligature around the descending aorta will raise the blood-pressure enormously in the still patent carotids, but will scarcely affect the pressure in the pulmonary artery. In fact the complete retention of blood in the left ventricle from closure of the aorta, raises the pulmonary blood-pressure only to about 3½ times its original value.

To produce pulmonary œdema, an immense increase in pulmonary blood-pressure is thus requisite. Similarly, it requires an enormous obstruction of the pulmonary veins to render the lung œdematous. Ligature of all pulmonary veins will produce œdema, but not always, since the circulation will sometimes cease before

œdema pulmonalis can occur. If, however, a small venous twig alone is left patent to carry the blood back to the heart, œdema is the invariable result. The same may also be obtained by compressing the left auricle, so as to obliterate its cavity almost completely.

These extreme conditions are, however, unlikely to occur in man. How are we, therefore, to explain the frequency of œdema of the lungs, one of the most frequent observations, post-mortem? If the left ventricle were unable to dispose of the blood sent by the right ventricle through the pulmonary vessels, a stasis would finally result in the lungs. This we would expect to occur when the left ventricle is paralyzed or at least weakened as compared with the right side of the heart. After long trials, Welch succeeded in producing this condition by compressing the left ventricle with his fingers. Whenever he succeeded in thus stopping the left side of the heart, while the right ventricle continued to pour its blood into the pulmonary vessels, pulmonary œdema was the inevitable result. Its occurrence in the human cadaver depends hence, on the earlier death of the left ventricle, while the right venrticle continues a few beats. The very speedy occurrence of œdema of the lungs is explicable by the delicacy and permeability of the pulmonary capillaries, especially under as high a blood-pressure as is produced by paralysis of the left ventricle.

Lesions of the Anterior Roots in Diphtheritic Paralysis.—*Gaz. Méd.*, 1877, No. 33. Déjerine examined post mortem three cases of diphtheritic spinal disease in children, in one of whom during life paralysis of almost all the muscles of the body had been observed in addition to the paralysis of the velum palati. In the other two cases a similar condition existed in the muscles of the upper extremities and neck. The anterior roots of the spinal nerves were placed in a one per cent. solution of hyperosmic acid for twenty-four hours, and then examined. In the first and most severe case Déjerine found in most of the nerve fibers the signs of a far advanced parenchymatous neuritis (degeneration). The axis cylinders were gone, the medulla fissured or infiltrated with drops of myeline; the nuclei of the nerve sheaths, as well as those of the intertubular connective tissue, were multiplied. Similar changes were observed in some peripheral nerves taken from the muscles; the muscular fibers themselves seemed entirely intact. Similar, though less marked, were the changes observed in the other two cases, in which the paralytic symptoms had been less extensive, and of shorter duration than in the first case. The changes in the nerves resembled those which appear when the nerves for any reason are deprived for a long period of the influence of their trophic centers, and it seems probable, according to Déjerine, that the changes in the peripheral nerves are dependent upon a cellular intra-medullary lesion. He promises the results of the examination of the cord at a future time.

